# Sexual abuse by superintending staff in the nineteenth-century lunatic asylum: medical practice, complaint and risk

**DOI:** 10.1177/0957154X20967299

**Published:** 2020-10-29

**Authors:** Cara Dobbing, Alannah Tomkins

**Affiliations:** University of Leicester, UK; Keele University, UK

**Keywords:** Great Britain, lunatic asylum, medical practice, patient complaint, sexual misconduct, trust, 19th century

## Abstract

The nineteenth century witnessed a great shift in how insanity was regarded and treated. Well documented is the emergence of psychiatry as a medical specialization and the role of lunatic asylums in the West. Unclear are the relationships between the heads of institutions and the individuals treated within them. This article uses two cases at either end of the nineteenth century to demonstrate sexual misdemeanours in sites of mental health care, and particularly how they were dealt with, both legally and in the press. They illustrate issues around cultures of complaint and the consequences of these for medical careers. Far from being representative, they highlight the need for further research into the doctor–patient relationship within asylums, and what happened when the boundaries were blurred.

Asylum provision in Britain during the nineteenth century expanded rapidly in ways that have been widely documented. Institutions have been evaluated as venues for the treatment or punishment of the insane, as instruments of social control, as an extension of welfare provision, and as evidence of social progress. Alongside this growth in infrastructure, the professionalization of ‘alienists’ was enhanced, not least because *ad hoc* responses to discrete establishments were replaced with a nationwide network of inspectors and an annual cycle of asylum visitation (8 & 9 Vict. c.100). Unsurprisingly, historiography concerning the development of psychiatry as a stand-alone profession charts the shift from its origins in the eighteenth century, when no formal training was required to take charge of the insane, to focus on the men at the head of the county lunatic institutions in the later nineteenth century (Scull, 1979; Shorter, 1997: 1; Smith, 1999). Asylums were critical in their careers, as they were the main site in which practical training occurred for the profession. There was a defined staff hierarchy in lunatic institutions, whereby formally recruited assistants to the superintendents gained knowledge and experience before progressing to the head of the asylum (Scull, 1976: 281–2). The increasing specialization of these practitioners meant that treatment for mental illness was given value as a distinct area of medicine. This process was assisted by the sharing of findings and ideas in contemporary journals such as the *Asylum Journal* established in 1853, and at annual conferences held by bodies such as the Association of Medical Officers of Asylum Hospitals for the Insane established in 1841. The latter, one of the first specialist sub-groups within British medicine, went on to form the Royal College of Psychiatrists (Bewley, 2008).

Little work has been done on the consequences of these developments for the balance of power between doctors and patients. The professionalization of asylum superintendents involved a consolidation of men’s authority; the expert status of doctors specializing in treating the mentally ill, the environment in which they practised, and the route for professional advancement had all been cemented by 1900, and doctors were in demand.

Did this mean that relationships within institutions for the mentally ill remained strictly professional, or became skewed by the power imbalance, despite inspection regimes put in place for patient protection? Or was the nascent profession of psychiatry in a very tenuous position at the close of the nineteenth century? Scull (1976: 287) contends that practitioner vulnerability was exacerbated, because although superintendents gained skills and responsibilities in managing populations of the insane, they did not discover practical solutions for curing them, which they had set out to do.

The expertise of asylum doctors meant that they held considerable power over their patients in terms of accommodation and treatment, where oversight was regular but not frequent. Concerns about the abuse of power were in circulation in the nineteenth century, and these tended to cohere around fears of wrongful confinement rather than exploitation of patients once under lock and key (Hervey, 1986). Protests from the patients themselves were heard by the Lunacy Commissioners, but investigations of patients’ complaints about staff members risked a loss of faith by both parties under examination: patient testimony was undercut by its coming from a mentally unstable source, while superintendents feared that Commissioner scrutiny gave legitimacy to complaints, no matter how far-fetched. Asylum case notes yield regular, if not very common, evidence of patients’ sexual stories which, if true, implicated staff in abusive regimes (Swartz, 2018; Tomkins, 2016). In most cases, and at this distance, it is not possible to judge the validity of accounts recorded as sexual fantasy.

This article gives sustained consideration to reporting of sexual contact between asylum doctors and their patients. It takes two case studies, one at each end of the nineteenth century, where sexual activity is testified by narratives beyond asylum notes. Both instances involved legal action in civil or criminal courts, allowing us to use them as a way to focus on the impact of the sector’s professionalization trends on doctors’ social relationships with both patients and their medical peers. The cases involve one private and one public asylum, the former in southern Ireland and the latter in northern England. Philip Myddleton and John Campbell are not regarded here as typical asylum doctors in any respect, but are instead used as touchstones for reactions to the abusive medical persona in 1816 and 1898, respectively. Their experiences illustrate, among other things, the instability of trust in medical men, changing cultures of complaint around medical practice, and the varying exertion of compliance and agency by vulnerable patients. The article concludes that patient exploitation could be egregious and with little risk to the doctor in the early nineteenth century, but that by the *fin de siècle*, professional regulation could operate very punitively upon the flawed, human practitioner.

## The asylum as social space and sexual opportunity

Asylums were opened as medical institutions and have been considered by historians as such. They have been less resolutely tied to understandings of the social lives of medical men. Since superintendents both worked and lived in their institutions, allowing the boundaries between these parts of their lives to blur or collapse, social relations on the premises could be central to men’s identity. Yet the relationships conducted by those in the psychiatric profession, be it with fellow doctors or with patients, are largely unknown beyond some rare exceptions. Digby’s (1985) study of the York Retreat does touch upon the familial relationship that occurred between the superintendent and the patients as a cornerstone of moral therapy, noting that the ‘patients were seen as dependents who needed to be looked after like young children’ (p. 59). However, the social constraint on former patients (except where they chose to expose wrong-doing) is matched by the reticence of medical men who rarely referred to social engagement with patients (beyond that sanctioned by formal asylum events such as dances) (Metcalf, 1818). Physical intimacy between doctors, co-workers and patients could feasibly inspire romantic or sexual contact, yet any sexual relations that may have taken place between doctors and others are absent from the historical literature.

Nor would contemporaries outside asylums necessarily have been aware of social or sexual contact across clinical boundaries, since such scrutiny as there was did not advertise findings of this sort. At the outset of the nineteenth century, British asylums were subject to minimal oversight (14 Geo III c.49).^1^ In England, the Metropolitan Lunacy Commissioners (active from 1828 to 1845) provided the model for a Commission covering all of England and Wales from 1845 (Bartlett, 1999: ch. 6; Jones, 1955: ch. 10), while the Lunacy (Scotland) Act of 1857 instituted a similar body in the General Board of Lunacy for Scotland (20 & 21 Vic c.71). Ireland instituted inspection of asylums in its Prisons (Ireland) Act of 1826 (7 Geo IV c.74), by making the inspector of prisons responsible for examining public and private madhouses; the Irish inspectors made annual returns from before 1844 (Inspectors-General, 1845). In this way, the conduct of most public institutions throughout Britain was eventually monitored by an effectual rolling survey, and complaints on either side of the doctor–patient divide could give rise to serious, specific investigation, but it is by no means clear that everyone wishing to protest was heard at this level. Complaints by subordinate voices may not have been heeded as at all compelling, as Sarah Wise (2012) has shown so dramatically. Certainly, historical sexual activity in asylums was not likely to be discovered by these means. Commissioners’ reports for England and Wales and the Board reports for Scotland up to 1899 reveal only one reported instance of ‘sexual intercourse’ (General Board of Commissions in Lunacy for Scotland, 1859).

Sex between doctors and emphatically vulnerable patients would have been questioned at any point in the nineteenth century, if revealed to public notice. Asylum inhabitants were by definition at risk on two counts: their mental disorder and their separation from friends and family beyond hospital walls. It is important to acknowledge, however, that legal protections could operate in contradictory ways. Violent sexual coercion was recognized decisively as a criminal offence, and rape risked incurring the death penalty up to 1841, which deterred juries from finding a guilty verdict. After 1841 the maximum sentence was life imprisonment, diminishing by the 1870s to five years in prison, if the act had been committed without violence. This eased juries’ consciences about finding defendants guilty, but evidentiary problems continued to inhibit the success of prosecutions. Rape as a legal term was problematic, as no statutory definition was recorded. As a result, uncertainty surrounded its use by magistrates, and a whole range of phrases were used to denote assault of a sexual nature. For instance, many assailants were charged with ‘indecent assault’, ‘carnal knowledge’ or ‘assault with the intent to ravish’ (Stevenson, 2010). Due to the nature of the act, it often bore no witnesses other than those involved, and resulted in one person’s word against another. Thus, with a lack of evidence, and with consent being difficult to ascertain, the conviction rate, as researched by Conley (1986: 521), was only 40 per cent, whereas for all other crimes it was 85 per cent. Rape accusations specifically against doctors were very rare until 1841, but occurred in some numbers across the remainder of the nineteenth century (Tomkins, 2017: ch. 5).

Asylum doctors were in daily contact with patients suffering from delusions, hallucinations or other symptoms which disrupted their charges’ perception of reality. The most acute social risks, then, arising from asylum medicine were that either a patient would convincingly accuse a practitioner of assault that had not taken place, or that a doctor would assault a patient and dismiss verbal or other evidence as a false product of the patient’s condition (Tomkins, 2016: 539). Both of these risks were realized during the nineteenth century, albeit exposed by different sorts of complaint. This article is in part a response to Reinarz and Wynter’s (2015) injunction for us to listen to complaints more attentively, and to this end we look beyond asylum case notes to two examples documented in court records and the press to learn more about the doctor–patient dynamic in such circumstances.

## Philip Parry Price and the scope for reinvention

The case of Philip Parry Price, who later styled himself Philip Parry Price Myddleton or Middleton, demonstrates the flexibility of practice open to men in the absence of a specialist field of medicine to deal with the mentally ill: psychiatry was in its earliest stages, and anyone, including non-medical people, could offer to take charge of vulnerable disordered patients. This case illustrates the flagrant abuse of trust that was possible in an unregulated medical profession which, despite widespread reporting, resulted in expressions of horror or disgust but no official sanction. Finally, it illustrates the protective social power of the occupational label ‘physician’ in the early nineteenth century.

Myddleton was one of three medical superintendents of the Hanover Park private lunatic asylum at Carlow in Ireland, established around May 1815. Private asylums were relatively new to the medical marketplace in Ireland, but their potential for perpetration of abuse quickly became apparent (Kelly, 2008a, 2008b). The asylum was exposed in the press as a scene of ‘medical pandemonium’ in 1816–17 when John Hinds, the husband of patient Hester Hinds, prosecuted Myddleton at the Court of Common Pleas in Dublin (Anon., 1816a, 1817).

It was alleged that Myddleton had ‘abused’ Mrs Hinds, who became pregnant. Mrs Hinds then ‘complained’ to Charles Delahoyde, another of the superintendents, who initially claimed he did not believe Hinds on account of her insanity. Eventually, after Myddleton did not deny the persistent accusations, Delahoyde confronted him, apparently to save the institution from public disgrace. Mrs Hinds was also fearful of social punishment. It was reported that she implored one of the men to ‘remove her to a place of secrecy and safety, where she might not be exposed’ (Anon., 1816b). Myddleton seems to have taken Mrs Hinds to the house of a Dr Rodgers of Wicklow, and installed her there under the assumed name of ‘Mrs Hamilton’, thus imposing on the ‘respectable’ Rodgers who allowed her to live as one of his family. She was delivered in Rodgers’ house and was subsequently visited by Myddleton (who treated her with evident affection).

Presumably the disordered or ostensibly compliant behaviour of Mrs Hinds encouraged Mr Hinds to bring the case as one of criminal conversation rather than rape. It would be difficult to determine whether Mr Hinds was acting on his wife’s or his own behalf, thereby showing women’s effectual or actual lack of power in the context of legal suits. However, his action may be seen in the early nineteenth-century context, insofar as he was making a stand against the violation of his ‘property’ – his wife. It could therefore be argued that he was acting on his outrage at another man having access to Mrs Hinds in a way that he did not, as prior to asylum committal they had separated. This could also be a sign of Mr Hinds’ embarrassment at his wife’s behaviour, as she was with other men at his expense, as he financed her care at Hanover.

The prosecution of Myddleton, and lengthy trial evidence, exposed the sexual promiscuity apparently exhibited by various members of asylum staff, including Delahoyde. Yet an apparently strong case from Mr Hinds’ legal team was dramatically undermined by the summing up of Lord Norbury, who concluded with the highly partisan instruction to the jury: ‘if you think he [Myddleton] can be acquitted, I shall be extremely well pleased’. Norbury usually found in favour of plaintiffs (Keane, 2004). Perhaps unsurprisingly, the jury could not agree on a verdict, and the case was dropped, with both parties paying their own costs. The medical men apparently returned to their occupations, at least temporarily, even though one or more of them had flagrantly broken the trust of their clients (patients and family members) while at Hanover Park. The case naturally raises questions about Myddleton’s background, training, professional demeanour and subsequent career.

Myddleton was baptized the son of Philip Price and Susan Parry in Clifford, Herefordshire in 1759.^2^ He was seemingly apprenticed to an apothecary in the city of Hereford, William Barrow, and took an apprentice of his own in 1785 (NA: IR 1/32, f. 1: SRO: D 1798/617/113). He married a relative (possibly daughter) of his former master and the couple had at least four children.^3^ The first indication of his larger ambitions came in 1791 when he acquired his physician’s diploma from St Andrews University (SRO: D 1798/617/113), and also published his *Treatise on the Diagnosis and Prognosis of Disease* (Price, 1791). This was the prelude to a decade of financial debt, misdealing, probable fraud and outright felony.

Time spent in America in the early 1790s, and the adoption of the additional surname Myddleton, facilitated a non-medical scheme for personal advancement. He devised a prospectus to attract skilled British craftsmen to join him in founding a colony in Kentucky, and even had silver tokens struck in anticipation of the need for his own colony’s coinage (Margolis, 1999). In doing so Myddleton ran foul of British law (22 Geo III c. 60) in the aftermath of the American War of Independence, since he was prosecuted for ‘seducing artificers’ to leave England for America (NA: TS 11/1122/5800; see also Anon., 1796). He was fined £500 and sentenced to one year’s imprisonment, but in the event remained in Newgate for three years before being transferred to the King’s Bench prison for non-payment of the fine (SRO: D 1798/617/113).

His eventual release from prison was achieved by his forming a new romantic alliance. While in King’s Bench he met Sarah Otto Baier, the estranged wife of Antiguan plantation owner Rowland Otto Baier (Oliver, 1894). By 1802 Myddleton was living with Sarah, two sons and Sarah’s daughters in Westminster, but the new family was still experiencing money trouble. They absconded with household goods after the Sherriff’s officer arrived to take possession, and moved to France (SRO: D 1798/617/113).

During the period 1792 to 1802, Myddleton had repeatedly induced people to trust him when there was no *prima facie* reason for them to do so. He extracted money from investors in the Kentucky project even while he was in Newgate; he paid debts in counterfeit coin; he delayed the loss of his goods in 1802 by claiming to the bailiff that he had been made a ‘privileged’ person by the Ambassador of Hesse Cassell (when he certainly was not).

Myddleton resurfaced in England in 1805 (SRO: D 1798/617/113; see also Anon., n.d.). He arrived in Stafford claiming acquaintance with Dr Francis Campbell of the Hereford Infirmary, brother of the recently deceased Dr Archibald Campbell of the Stafford Infirmary. He affected to be a man of property to the tune of £2000 a year and not to care about medical practice: even so, on the basis of the connection, he persuaded Archibald’s widow to take him as a tenant and introduce him to her ‘friends’. Myddleton quickly sought to establish himself as a medical practitioner. He was ‘generally visited by the inhabitants of Stafford’ and was elected to the Stafford Infirmary as an honorary physician.

Opinion in Stafford swung against him, and hardened when Myddleton decided to prosecute surgeon Francis Hughes for slander. He based his case on the notion that Hughes was motivated by a desire to remove a competitor in the medical marketplace, or ‘malice set on foot by a mercenary motive’ (Anon., n.d.: 12). This may have been accurate, but Hughes’s words (relating to Myddleton’s departure from Westminster, with household goods that were technically being held for debt) were substantially true. Myddleton lost the slander case, spent a fortnight in Stafford gaol for a long-standing debt, and then withdrew from the town, only to reappear in Ireland around 1812 (Kelly, 2016: 51–2).

In the years after the Dublin court case, Myddleton continued to practise medicine. He achieved a high level of personal agency, at least in part by using his physician status to good effect. He travelled back to America and resumed his medical career by (among other things) lecturing on pulmonary disorders (Anon., 1824). Eventually he returned to Bath in England where he produced two further publications: *A Preliminary Dissertation Illustrative of a New System of Pulmonary Pathology* (1825) and *An Essay on Gout* (1827). The latter was reviewed seriously, if sceptically, in *The Lancet* (Anon., 1827). His final move was to Heavitree in Exeter, where he died in 1830 (Anon., 1828).^4^

Myddleton’s creative capacity for reinvention seems to have been undiminished, despite repeated public ‘complaint’ in the form of penal and social punishments, and his efforts were willingly supported by communities in both Britain and America (until and unless his behaviour was discovered). His acquisition of the label ‘physician’ arguably became an important factor in his continued agency. Myddleton could clearly appal his contemporaries, but was allowed to continue with his personal and professional life by dint of repeated relocation. This freedom of action was of its time; by the date of his death, structural changes to the legislative and professional landscapes were altering the way that complaints about medicine were channelled.

## Evolving cultures of complaint and making examples

Myddleton’s contemporaries who were dismayed or materially disadvantaged by his behaviour had no clear course of action open to them. The newspaper and the courtroom offered sites of reproach and a measure of restitution, but only in as far as the wronged parties were prepared to wrest these for themselves. Myddleton was only subjected to attention by the state when his actions bordered on treasonous; the ‘repertoire of contention’ was very limited (Tilly, 2006: 39). Nor was there, outside medicine, a culture of complaint directed to medicine that could readily recognize Myddleton’s wrong-doing. Practitioners were traditionally the butt of the joke in visual satires that dwelled upon their age, short-sightedness, self-absorption or corpulence, not on their potential for criminal offence (Vincent, 2005). The stereotype of the unfeeling ‘sawbones’ was probably grossly unfair, but it was nonetheless in wide circulation (Brown, 2017).

In relation to a private asylum operating in Ireland in the 1810s, there was no clear site for the reception of a complaint, albeit regulatory regimes are more reliably built up around institutions or sectors than around problematic individuals (Reinarz and Wynter, 2015: 7). Within the profession, quackery (however construed) was the target of much orthodox ire, but Myddleton did not fit the customary targets of this narrative either. He held a recognized qualification and did not sell ‘cures’ in the way most recognizable to early nineteenth-century peers (Porter, 2001: 128–9). His co-option of Delahoyde’s treatment for the insane begins to look akin to the sorts of startlingly-effective treatments sold by empiric specialists, but few of the latter could boast a published report as evidence for the endorsement of royalty. There was no ideological space for acknowledgement and chastisement of a man like Myddleton.

Statutory precedent for England came in advance of Myddleton’s wrong-doing, but effective legal action remained lacking. An Act of 1774 required the proprietors of provincial asylums in England to seek licensing from the magistracy, and submit to annual inspections (14 Geo III c.49). Justices made visits thereafter, although whether they were yearly or more sporadic depended on local energies (Porter, 1987: 152). No prosecutions were brought as a result of the Act, however, so that complaint effectively remained with the conscience of the visiting magistrate. Furthermore, the law did not apply to Ireland.

This context for medical misconduct in Britain changed radically over the course of the nineteenth century, in the confidence to identify exploitative behaviour, the impetus to complain among different social groups, the fora for examining complaint, and the penalties that could legitimately be sought. The appetite for scrutiny came first from within the medical profession. The impetus of medical ‘reform’ inspired, among other developments, the founding of *The Lancet* and the related exposure of ignorant or unskilled practitioners by their more confident peers (Jones, 2009). These intra-professional moves were supported by foundational legislation. An Act of 1828 founded the Metropolitan Commissioners in Lunacy, and the first intervention with widespread geographical application followed quickly with the inauguration of the reformed poor laws (7 Geo IV c.74; 4 & 5 Wm IV c.76). Price (2015: 4–5) has forcefully brought our attention to the New Poor Law of 1834, and the scope it offered to Guardians of the Poor, non-medical relieving officers, and paupers to protest about medicine.

Complaint ‘is a reckoning with the past’ (Reinarz and Wynter, 2015: 2). It is also a means of future governance where a problem generates its own cautionary example. In the early part of the century, the negative exemplar had limited force, as the anger of one practitioner towards another could be written off as a function of competition for patients, therapeutic difference, or another one-off conflict. There were no widespread public calls for restitution from beyond medicine, targeting either orthodox or marginal practice, and no pillorying of men beyond local audiences.

The inquest was a venue for testing medical authority throughout the nineteenth century: its part in the culture of scrutiny and critique of medicine fluctuated. After Thomas Wakeley began campaigning for medical coroners, the stakes were raised for practitioners as witnesses as well (Burney, 2000: 107–64). Inquests were potential hazards for doctors who could be seen as scapegoats in the absence of an obvious, malicious actor. Even so, the ground shifted in unexpected ways in the 1850s with an inquest on a patient of Dr William Palmer leading to his execution for murder. The deaths attributed to Palmer’s poisonous attendance had long-term consequences for the status of forensic medicine (Burney, 2006: ch. 5). They also had an attenuated impact on the perception of the medical profession. At first the occupation of medicine did not suffer too badly: Palmer was held up as the exception who proved the rule of typical medical probity. As time passed, the occasional emergence of other men like Palmer contributed to an undercurrent of wariness about medical power (Tomkins, 2017: 176).

Medical control over the human body was undoubtedly on the rise between the 1840s and 1870s, not least because of the introduction of anaesthetics and antiseptic practice to surgery. Even so, the expansion of power did not give rise to a relaxation of professional concern about complaint. Rather, towards the end of the century, the profession increasingly tried for foreclose on all but unavoidable criticism, particularly when it came from non-medical critics (e.g. Tomkins, 2017: 137, 258). At the same time, perhaps in partial response to the internal nature of legitimate medical questioning, the public attitude to medicine combined a rising respect with a lurking suspicion. By making medicine powerful, the profession also made it unknowable by the general population (and imagination filled the gap). The publication of *The Strange Case of Dr Jekyll and Mr Hyde* in 1886 followed by the Whitechapel murders in autumn 1888, for example, provided a medical model for the identity of the murderer: the physician Hyde and Jack the Ripper could legitimately be regarded as ‘two and the same’ (Winter, 2015).

As a result of these developments, ideological and statutory apparatus for complaints about medicine had been transformed by the 1890s. Current research suggests that both the expanding capacity for complaint in the first half of the nineteenth century, and the contracting acknowledgement of justified complaint in the second half, were both most palpable within the profession, and somewhat at odds with the pace of changing sentiments among the lay public. A second emblematic sexual scandal, again featuring a practitioner having sex with an asylum patient, shows how far the culture of complaint had evolved. John Campbell, unlike Philip Myddleton, was not given the space to reinvent himself: instead, his ‘self’ became subsumed into exemplary punishment and permanent loss of liberty, resulting in a different form of injustice. In his case, complaint as a call to action became an over-reaction.

## John Campbell and the pathology of error^5^

The case of Dr John Campbell offers a contrasting example of how sexual relations between doctor and patient were regarded in the nineteenth century, albeit 82 years after Myddleton’s misdemeanours. Much less prolific in wrong-doing, Campbell was a respected and celebrated asylum superintendent, who dedicated his life to treating pauper insanity. His sexual exploitation of a female patient in 1898 came as a great shock to the surrounding community, and signalled the end of his career.

The increased oversight of the profession is clear when comparing his career to Myddleton’s. In the latter half of the nineteenth century, the superintendent role was rigidly presided over by the Lunacy Commissioners. Any misdemeanours or malpractice that flouted the Lunacy Act could result in prosecution, staff being sacked or the imposition of a fine. Career progression was also much clearer and less *ad hoc* than earlier in the century. Even so, no specialist training was required, and all experience of insanity was gathered on the job. The asylum therefore became a theatre for training and development for the new medical superintendents who, with a growing number of psychiatric journals, also shared their experiences and approaches to mental treatment (McCrae and Nolan, 2016: 16).

The role was extremely demanding. Superintendents resided in the grounds of their institutions, and were constantly on call. A typical day lasted twelve hours of inspecting the wards, writing reports and, in the afternoons and evenings, attending social functions associated with the wider psychiatric profession (Russell, 1988: 304–5). The physical and, specifically, mental strain of superintendence was such that in 1860 officers were urged to be careful of their own mental health, and in the subsequent historiography have been presumed likely to suffer depression or neurasthenia (Oppenheim, 1991: 153–4).

Campbell came to prominence in the still nascent field of psychiatry as the superintendent of the Garlands Lunatic Asylum, Carlisle (1873–98). This was reinforced by the dozens of articles he published on his work and research carried out at Garlands. This high standing, and apparent excellent record, was undermined by his assault of a female patient, three weeks before he was due to retire. On 11 August 1898, he was seen by a laundry-maid taking a female patient, Janet Mooney, into a coal cellar and having sex with her. Unusually for most allegations of serious sexual assault, there was no room for doubt: the door to the cellar was left open, and intercourse was subsequently witnessed by other servants. The act was labelled as criminal misconduct, and the implied consent of the patient, as she had accepted payment of 2d from Campbell, did not affect the potential for the maximum sentence of two years imprisonment. The incident was clear abuse of his power, and in breach of the 1890 Lunacy Act, so the Commissioners had ‘no option’ but to prosecute (Anon., 1898d).

All witnesses commented that Campbell was in a state of intoxication while committing the act, and was not his usual self. Immediately, the Committee of Visitors was informed and an investigation ensued. In the following days, Campbell threatened suicide through laudanum overdose, and was put under constant supervision at his home, with an attendant being specifically instructed to remove all his razors. However, he soon forgot the idea of suicide when he realized that if he took his own life, his life insurance would not be paid out to his family (CAC: C/C/2/417).

The trial at Carlisle assizes in November involved evidence from several of his colleagues. Interestingly, those who worked closest to him commented that he had been an alcoholic for some time. Dr Farquharson, who had been his assistant for many years, stated with confidence that Campbell had been intemperate since August 1892, and from late 1897 the problem had worsened. This situation was severely criticized by the judge who showed outrage at how ‘a man who, according to a number of eminent doctors, superintendents of important asylums, showed signs of madness, months and even years before, was allowed to go on without report and observation as superintendent’ (Anon., 1898a).^6^ Thomas Horrocks, chair of the Asylum’s Visiting Committee, stated that he knew nothing of Campbell’s intemperance, and defended him by stating that ‘any person who had been so long in the position he had occupied had been there quite long enough’. Although there were rigid inspections – a departure from earlier in the century – they had failed to hold inadequate staff to account.

The evidence also brought to light Campbell’s boastful fabrications of his achievements. For instance, he insisted that one of his clients was a member of the royal family. He also boasted that he was moving to London to treat wealthy clients for a higher fee, and was going to set up his own practice in Harley Street (CAC: C/C/2/417). These grandiose notions of his own position may indicate his professional desires for advancement, which he had not been able to realize; they are certainly characteristic of other practitioners who suffered poor mental health around the same time (Tomkins, 2017: 215–16). Campbell had published over 40 articles in leading medical journals, and had become president of the Border Counties Branch of the British Medical Association, but we must assume he felt he had fallen short of his own ambitions (Digby, 1994: 174). A glimpse of this self-deprecating view of his abilities can be seen in his 1896 address to the Section of Psychology at the Annual meeting of the British Medical Association, held in Carlisle. Despite his numerous accolades, Campbell down-played his expertise, and alluded at the end of his speech to ‘the many topics which I have hurriedly, lightly and imperfectly touched on’ (Campbell, 1896: 27). Thus he indicated, albeit in a muted and normalized way, his sense of vulnerability regarding his own professional position.

Campbell was found guilty but insane at the time of the assault, and was committed to Broadmoor as a criminal lunatic (Anon., 1898c). The testimony from close colleagues seemed to paint him as a victim of mental illness and, in order to address his condition publicly, the authorities had to detain him in the correct place so that his misdemeanour was seen to be dealt with. Campbell’s lawyer argued that the correct place to confine him would be a lunatic asylum, rather than in prison, to save his reputation (Anon., 1898b). A letter dated three days before his removal to Broadmoor stated that he was ‘very anxious to know if he will be allowed to wear private clothing’ (BRO: D/H14/D2/2/1/1798). Arguably, this demonstrates that he still felt he was above the criminal classes with whom he was to be housed.

Despite remaining abstinent from alcohol throughout his time at Broadmoor, Campbell retained his inflated sense of achievement. For example on 1 March 1900 it was recorded:

He has no symptoms of actual insanity, but he is very conceited, egotistical and boastful, and has an exaggerated opinion of his own capabilities and importance. He would be very liable to relapse if he again indulged in alcohol and I am exceedingly doubtful if he would continue temperate if discharged.

Campbell remained in Broadmoor until 28 May 1900, and was transferred to High Shot House, a private home for inebriates, where he continued to serve the conditions of his sentence under the care of Dr Albert Neale (BRO: D/H14/D2/2/1/1798).

Taking into account Campbell’s abuse of his position of power, and subsequent incarceration as a criminal lunatic, the way in which he was regarded in the years surrounding the incident is quite astounding. In initial newspaper reports, there was immediate shock, but there was little mention of the victim, and emphasis was continually placed on Campbell’s contribution to furthering the understanding of insanity. For instance, a report in the *West Cumberland Pacquet* (Anon., 1898d) provided detailed evidence of the assault, with accompanying witness testimonies, but deemed it to be ‘painful rumours’. The report described Campbell as having ‘done much good service for the two counties of Cumberland and Westmorland’, and that the Lunacy Committee expressed ‘high appreciation of his work’. This seems somewhat remarkable for an article which provides detailed accounts of the assault, and even specified the exact clause of the 1890 Lunacy Act he had violated.

Considering the nature of his misdemeanour and the perceived progression in the accountability of the profession, it is surprising that the ostensible, public reputation of such men remained intact. The difference between the early and late nineteenth-century modes of dealing with such ‘misdemeanours’ was that the latter were more regimented, so that, in the public arena, the authorities could be seen to be actively addressing the issue. In Campbell’s case, the penalty was imprisonment, but as he was deemed to be insane at the time of the crime, he was permitted to serve his sentence in an asylum. Professionally, he was technically unaffected. Although he never practised medicine again, Campbell remained on the medical register until his death, which is evidence that the General Medical Council was an imperfect prosecutor of wrongdoings by practitioners into the twentieth century. The key difference between the two cases of Myddleton and Campbell is that Campbell was not ostracized at any point: rather, he was treated as a victim of alcoholic insanity caused by the pressures of the profession. The exposure of Campbell’s assault led to the end of his career, upheld by the increasing rigidity imposed by the Lunacy Laws, but did not damage his reputation or his claims to an intellectual legacy.

## Implications

Myddleton and Campbell, chosen here as emblematic cases demonstrating the changing response to the misdemeanours of practitioners in charge of the mentally unwell in the nineteenth century, raise some important points of consistency and divergence. Both men abused their positions of power in order to seek sexual gratification, and broke the trust instilled in them to care for the vulnerable. The two cases demonstrate the persistently patriarchal welfare system that presided over those certified insane. Although the rigid inspection of mental institutions increased during the nineteenth century, the fact remained that patients were superintended by men, and treatment was prescribed by male doctors. This had the potential to yield unfortunate implications for female patients residing in institutions, who had little scope for choice and negotiation when confronted with sexual exploitation (e.g. Hide, 2018).

Regarding the risk of prosecution for sexual crime, and the consequences of conviction, our two cases both highlight the importance of consent. Myddleton, and it was suggested some of the other Hanover Park doctors, was perceived to have had consent for sexual relations with Mrs Hinds. The allegation that she held frequent liaisons with several men while in an asylum served against her character and deemed her to be a consenting party, despite her illness. As Stevenson (2010: 83–4) comments, the law failed to stipulate what consent was, and unless it was clear that the victim resisted the act, through violence, threats or force, then consent was assumed in all cases. Therefore Hester Hinds, despite her complaints of sexual abuse, was regarded as a consenting party, which diminished the case against Myddleton.

Consent is also a significant if mutable factor in the 1898 case. The circumstances of Campbell’s exposure meant he never risked being charged with rape, but even so the nature of his conviction threatened his respectability much more than similar circumstances had injured Myddleton’s. The key factor was the witness testimonies. As the act had taken place in public, and had been viewed by several people, he was in no position to deny the assault. Alongside this is the fact that, since 1845, the Commissioners in Lunacy (a body that did not exist in Myddleton’s era) could prosecute anyone who broke the Lunacy Act. They were proactive in bringing to account those who flouted the law in ways that could be proved, including superintendents, and were rigid in upholding it. A common violation was that of detaining insane individuals without the proper order and certificates, but more serious crimes can be found, such as causing death by inflicting violence on a patient.^7^

That said, it is evident in the trial statements that the Lunacy Commissioners and Campbell’s colleagues seem regretful that he was caught in the act. It is plausible that, had it happened in private or if there was only one witness, then he would have retired at the end of August 1898 unscathed, and the whole event would have been dealt with out of the public gaze. In this period, a respectable gentleman in an important position, such as Campbell’s, would ask eminent colleagues to corroborate his reputation, which occurred in the trial. However, respectability would be lost if successfully convicted (Stevenson, 2010: 91). The only factor preventing him being placed in a prison with common criminals was his apparent alcoholic insanity.

Analysing the two cases in terms of the implications of sexual assault highlights the importance of compliance among medical peers. Both Myddleton and Campbell had colleagues who colluded with them in their misdemeanours. This was more pronounced in Hanover Park, as there was shared institutional responsibility among three superintendents. Evidence reported in Myddleton’s trial strongly implied that all the doctors were partaking in relations with patients, and even with Mrs Hinds. The collusion continued when it came to light that she was pregnant and was transferred to conceal the birth. The doctors pretended that she had been removed by friends, and it was only when Delahoyde spoke honestly that her husband found out the truth. For Campbell, compliance is evident through the numerous colleagues that stated he had been an alcoholic for several years. They had not brought this to the attention of either the Commissioners or the Committee of Visitors, and it ultimately manifested in a worsening of his condition, and to the assault on Janet Mooney. Thus, collusion and compliance in both instances added weight to the implications of the acts of sexual exploitation that came to light.

Both cases provide a potent indicator of how female victims were treated in both periods, as their voices remain largely hidden: we do get glimpses of Mrs Hinds from the reports of Myddleton’s case, but any real sense of her experience is as unclear as that of Janet Mooney. Hester was of higher status than Janet, as she was in a private, fee-paying institution funded by her husband, as opposed to a pauper in a public asylum. Therefore, we may expect the earlier legal case to have been successful, rather than the later one. This may suggest that the cases were less about the female victims, and more about the reputation of the bodies presiding over the profession of the perpetrators.

By the later decades of the nineteenth century, the complaining patients were listened to in a more structured fashion, as they could notify doctors, and any official visitors of their grievances. Ironically, in 1881 the *Journal of Mental Science* published Campbell’s address to the Medico-Psychological Society: ‘Complaints by insane patients.’ In this he states that ‘no one portion of duty is more unpleasant than having to listen to complaints of ill-treatment’. Nevertheless, they were listened to. Campbell outlines the procedure by which complaints of personal violence are investigated when received at Garlands: ‘to have the patient stripped and examined . . . [which] ensures the safety of the patient, and necessitates care and accuracy in reporting of injuries by attendants as soon as they know that such matters cannot be hidden’ (Campbell, 1881: 350). However, he also recommends caution in believing every complaint, as they were often ‘the result of delusion’, and relays the case of a female who ‘complains to me each morning that the female attendants are men in women’s clothes, and that they “raped her” during the night’ (p. 348). Expressions of agency, therefore, through patients speaking out about mistreatment, were regarded with more apprehension than at the beginning of the century, albeit with no guarantee of effective redress.

In Campbell’s criminal misconduct, agency was not demonstrated by the patient who was assaulted – unless we consider her receipt of payment as a form of agency; instead, it can be imputed to the laundry-maids who witnessed the event. As mentioned above, the success rate for convictions of sexual crime depended on the ‘connection’ having witnesses or being corroborated by more than one party. The crucial testimonies in Campbell’s case came from the lay members of staff who observed the act. The female attendants were the lowest paid, and were the least powerful of all the staff (McCrae and Nolan, 2016: 29–30). There had been notable cases of reported sexual relations between asylum staff members, brought to light by women, that had not been believed. The men had denied it and there had been no witnesses, as mirrored in most sexual misdemeanour accounts.^8^ Therefore, the potential for these women not to be believed was high. However, as Campbell’s assault took place in such a visible place, the female staff had to report it, and stood a great chance of being believed as there was so much corroborating evidence. The fact that Campbell left the door open is significant, and raises some important questions – did he want to be found out? His alcoholism had not attracted any significant attention, was it that he had lost hope by this point (it was noted in the trial documents that his wife and daughter were not now living with him) – and did he therefore not care if he was found? Or did he have great faith in his position of power? Did he think that he was untouchable, and assumed the maids would not speak out, or that they would not be believed? These unanswerable questions have implications in terms of Campbell’s motivations for the act, which indicate the fragility of his mental health by August 1898.

Vulnerability, then, is another common thread in both Myddleton’s and Campbell’s stories, affecting themselves as well as their victims. Myddleton’s constant need for reinvention was arguably driven by his sense of social, professional or financial vulnerability. Regular name changes, several marriages, relocation, professional reinvention, and stretches in gaol, all added to his constant precarity. In contrast, his patients at Hanover Park were in a medically vulnerable position. Myddleton was in a position of trust, and held power over those under his care, particularly as they were institutionalized and could not discharge themselves. As Pellegrino (1985: 15–16) reminds us, patients trust physicians because they have been disabled by illness and are dependent on them for care. This unequal relationship results in extreme vulnerability on the part of the patients, which Myddleton exploited for his own pleasure.

Despite Campbell’s solid professional position, as compared with Myddleton’s tenuous one, his vulnerability is demonstrated by his boastful, grandiose notions of self-importance. Being unable to effectively treat a large proportion of his patients would have also added to his own mental fragility, as he openly hinted at in a letter published in 1897 in the *Lancet* entitled: ‘The hardships and risks of the medical profession and those engaged in the treatment of bodily and mental disease’. He alluded greatly to the strain caused by his job: ‘The mental anxiety experienced by all in the medical profession is at times almost excessive’ (Campbell, 1897). The isolation and segregation of living on the fringes of the local community, the demanding, unrelenting hours, the budget constraints imposed by the Asylum Committee, the constant institutional overcrowding, and the concentration of hundreds of unpredictable individuals all added to the fragility of Campbell’s position (Hide, 2014: 46–7).

A consequence of vulnerability for Campbell was alcoholism. His addiction rendered him incapable of serving his sentence in prison. Although he lost respectability by being found guilty, he retained an element of dignity, and was portrayed as a victim of his illness that had been brought on by his role. Campbell was familiar with treating alcoholic insanity in Garlands, and the admission, particularly of male inebriates, was common (CAC: THOS 8/1/3/1/10: 16). Typically, they were relatively easy to treat, as the asylum did not allow alcohol of any kind in the ordinary diet, so the patients would sober up quite soon after committal. Taking this into account, Campbell’s condition after conviction is complicated by the fact that when he stopped drinking he was no longer considered decisively insane, but his grandiose ideas of himself remained. This could have been a consequence of habitual drinking over a prolonged period which, according to Victorian medicine, could have irreversible effects on a man’s health. The ‘bestiality’ of the intoxicated male was also emphasized, which may help to explain, in part, the ‘victim’ rhetoric evident in the reports of Campbell’s assault: that he was not to blame – rather, it was the alcohol (Makras, 2015: 139–40).

In the final analysis, neither of the men was banned outright from practising medicine after the sexual exploitation. This returns us to the notion that the role of physician was protected by his position in society. This is clearer, however, in Myddleton’s case, as he continued to practise actively, and the trial lapsed without resolution, whereas the prevailing portrayal of Campbell as a victim is extremely telling of the way in which the form of sexual assault was regarded, and his medical status was maintained only on paper.

## Conclusions

Further work into superintendents as people, and their relationship with their patients, is clearly required to create a comprehensive picture of the social implications of delivering care in asylums. The challenge remains that surviving sources are predominantly medical documents written by doctors. Thus, delusions of patients involving sexual advances, and abuse, are largely consigned to history as fabrications and indications of illness, as we have a limited capacity for discerning the truth. The two emblematic cases detailed here go some way to address this gap in the literature, but, most importantly, this study highlights the need for further research, as little is understood of the patient–doctor dynamic in institutions that have been the subject of large-scale focus.

Myddleton and Campbell were born 85 years apart, and their medical careers were subject to very different expectations and contexts. As demonstrated, there are some commonalities between their cases beyond the outline narrative of sexual exploitation of patients in their own asylums. Even so, the divergent reactions to the two men at the heart of these accounts are highly instructive. In moral terms, Myddleton may have been the more egregious, if only in his extended attempts to conceal his abuse, but he was subjected to a civil trial; in contrast, Campbell underwent a criminal trial. It is not surprising that Campbell’s case attracted more widespread attention, given the increased scope for oversight of both practitioners and asylums across Britain by the 1890s, but the attention was of a very particular kind that foreclosed on Campbell’s future. Myddleton literally walked away from most of his misdemeanours and crimes, and enjoyed scope for repeated reinvention, whereas Campbell was not allowed to try to ‘start again’, even as a retiree. Instead, he was subject to a form of confinement for the rest of his life, owing to his peers’ alarm and concern. The structures for the containment of social damage to the profession were not present in 1816. The apparatus of regulation present in 1898 was used without recourse to the GMC, but was reinforced spontaneously for the occasion. The deployment of Broadmoor (from which discharges were probably easier than from prison) and understandings of alcohol addiction were both regretfully pressed into service.

This disparity was not determined solely by material provisions (such as the building of a criminal lunatic asylum) and secure professional regulation. Rather, it is symbolic of the ongoing definition of professional behavioural boundaries. Responses to doctors were largely respectful throughout the nineteenth century, but the consequences of that respect when expressed by patients or colleagues altered significantly over time. This research suggests that in the early nineteenth century respect was conferred repeatedly and that this favour was only withdrawn when wholly inescapable reasons emerged to challenge or overwrite it. The damage inflicted by discovery remained discrete to the practitioner and could be evaded by geographical removal. By the later nineteenth century, exposure of wrong-doing inspired wariness and concern, such that the practitioner was not castigated by either colleagues or the public but handled firmly, sorrowfully and gently, ultimately as a victim rather than a perpetrator. Deviance in the medical profession had become so aberrant as to deserve special pleading, lest the errors of one man became damaging to all.

## Notes

1. This and similar references refer to Acts of Parliament.

2. Baptism of 16 April 1759; his parents were married in the city of Hereford on 25 October 1751.

3. Baptisms at All Saints parish, Hereford: 8 May 1785, 31 May 1786, 14 May 1787 and 19 August 1788.

4. Burial 11 September 1830 at St Sidwell’s parish, Exeter.

5. Some details of Campbell’s prosecution have also been published in an earlier article by the author: Dobbing, 2016: 9–10.

6. Alcoholism was a common reason for asylum admissions in this period; Makras, 2015: 145.

7. For an example of the latter, see the case of William Wilson in 1870, who died after being punched and kicked by two attendants at Lancaster Asylum; they were prosecuted and sentenced to seven years penal servitude each; Commissioners in Lunacy, 1870: 17–18.

8. For example, a laundry-maid of Caterham Asylum placed a summons against the asylum reverend for maintenance for their illegitimate child, but the case was dismissed; Anon., 1893.
